# Supplementary material for: Glacial melt disturbance shifts community metabolism of an Antarctic seafloor ecosystem from net autotrophy to heterotrophy
Source: Commun Biol. 2021 Jan 29;4:148. doi: 10.1038/s42003-021-01673-6 (PMC7846736; doi:10.1038/s42003-021-01673-6)
Supplement: Supplementary file 2 — Supplemental Information [file 42003_2021_1673_MOESM2_ESM.pdf]

Supporting information for:

Glacial melt disturbance shifts community metabolism of an Antarctic seafloor ecosystem from net autotrophy to heterotrophy

Ulrike Braeckman*, Francesca Pasotti, Ralf Hoffmann, Susana Vázquez, Angela Wulff, Irene R Schloss, Ulrike Falk, Dolores Deregibus, Nene Lefaible, Anders Torstensson, Adil Al-Handal, Frank Wenzhöfer, Ann Vanreusel

*corresponding author: phone: +32 9 264 85 34 - Ulrike.Braeckman@UGent.be

Tables

Supplementary Table 1: Location, water depth, and date of sampling of the three sites in Potter Cove

Site		Faro	Isla D	Creek
Latitude		62° 13.31' S	62° 13.30' S	62° 14.08' S
Longitude		58° 39.36' W	58° 38.30' W	58° 39.43' W
Water depth [m]		8–9	6–7	8–9
In situ measurements and sampling for biogenic compounds (Dates)	summer 2015	10–12/02/2015	18–19/02/2015	28/02–01/03/2015
	winter 2015	28–29/09/2015	12–13/09/2015	-
	spring 2015	29–30/12/2015	22–23/12/2015	14–15/12/2015
	spring 2016	12–13/12/2016	19–20/12/2016	01–02/12/2016

Supplementary Table 2: Spatio-temporal variability in benthic biotic parameters. Significant results from two-way ANOVA or permanova analysis and subsequent Tukey pair-wise tests. Df num, denom refers to degrees of freedom numerator and denominator.

Response variable	Test	Transformation	Factor	F-value	df num, denom	Comparisons from Tukey HSD test with $p < 0.05$
chl-*a*:CPE ratio 0-1cm	Two-way ANOVA	-	Site:Season	32.15***	10, 33	spring 2015 > all other seasons<br>spring 2016: Faro > Isla D<br>summer 2015 > winter 2015
Benthic microalgal biomass 0-0.5 cm	Two-way ANOVA	log10	Site:Season	10.54***	10, 26	spring 2015: Faro, Creek > Isla D<br>Faro: spring 2015 > all other seasons<br>Isla D: spring 2016 > winter 2015
Prokaryotic biomass 0-5cm	Two-way ANOVA	-	Site:Season	7.33***	10, 26	spring15: Creek > Isla D<br>spring 2016: Faro > Isla D
Meiobenthic biomass 0-5cm	Two-way ANOVA	-	Site:Season	6.61***	10, 32	summer 2015: Isla D > Creek<br>winter 2015: Isla D > Faro<br>Faro: spring 2015 > winter 2015 and spring 2016
Laternula elliptica biomass	Permanova	-	Site:Season	21.06*** (pseudo-F)	4, 128	Faro: summer 2015 > spring 2016<br>Isla D: summer 2015 and spring 2016 > spring 2015<br>Creek: summer 2015 > spring 2015 and 2016<br>summer 2015 and spring 2016: Isla D > Creek and Faro
Macrofauna biomass excl. *L. elliptica*	Two-way ANOVA	-	Site:Season	2.34°	8, 24	-
			Site	4.03*	2, 28	Isla D < Creek
			Season	5.47*	2, 28	summer 2015 > spring 2016

*** $p < 0.001$; ** $p < 0.01$; * $p < 0.05$; ° $p = 0.051$

Supplementary Table 3: Sediment characteristics of the three study sites, averaged over the upper 5 cm. Means ± se. N = number of samples; MGS = Median Grain Size; TOC = Total Organic Carbon; TN = Total Nitrogen. Source data can be found in Supplementary Dataset 7.

Site	N	MGS [µm]	% silt	TOC [%]	TN [%]
Faro	62	102 ± 5	42 ± 1	0.23 ± 0.01	0.063 ± 0.003
Isla D	66	18 ± 2	85 ± 1	0.22 ± 0.01	0.055 ± 0.003
Creek	49	92 ± 4	39 ± 1	0.34 ± 0.02	0.059 ± 0.004

Supplementary Table 4: Significant models resulting from regression analysis. Models only include significant (p < 0.05) partial regression coefficients. NCM = Net Community Metabolism; SPM_w = Suspended Particulate Matter in the water column. Chl-*a*:CPE = chl-*a*:Chloroplastic Pigment Equivalent ratio. Source data can be found in Supplementary Dataset 3.

	Model	N	R^2_{adj}	*p*-value
NCM	NCM = -35 -1.26 x SPM_w + 0.73 x chl-*a*:CPE	30	0.67	< 0.001
chl-*a*:CPE	chl-*a*:CPE = 47+ 0.18 x Microalgal biomass	37	0.32	< 0.001

Figures

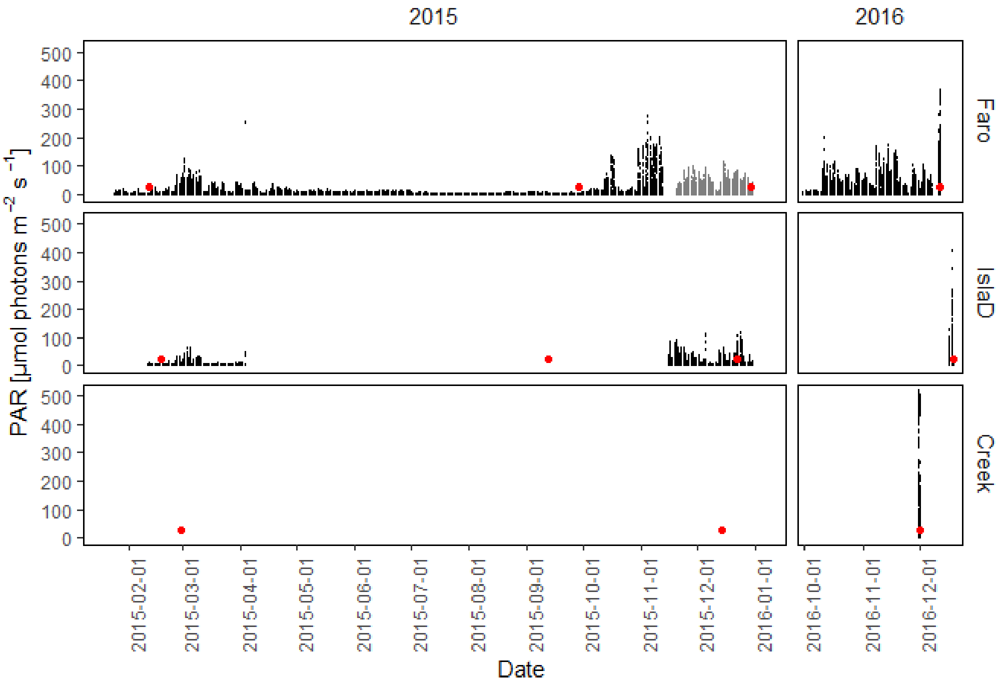

Supplementary Figure 1: Photosynthetic Active Radiation (PAR) measured by Odyssey PAR-sensors (Odyssey Photosynthetic Irradiance Recording System, Data Flow Systems, Christchurch, New Zealand) installed approximately 0.5 m above the seafloor at approx. 10 m water depth at Faro and Isla D throughout 2015 and November-December 2016. Data for Isla D are only sparsely available since ice scouring frequently damaged the light sensors close to the glacier. Also at Faro, there was a lack of data in December 2015. The grey data points are from a location at ~500m distance from Faro at 11 m water depth. These PAR-sensors were calibrated according to Deregibus et al. (2016) and PAR was measured with a temporal resolution of 30 minutes. At Creek, PAR was measured only during the spring 2016 incubation, a Li-Cor PAR sensor (LI-192, Li-Cor Biosciences, Lincoln, Nebraska, USA; factory calibrated) with a temporal resolution of 1 s for 36 h. Red dots indicate the date of the in situ incubations and the experimentally determined light compensation point (26 µmol m^{-2} s^{-1}) (see methodology in Hoffmann et al. 2019). Source data can be found in Supplementary Dataset 4.

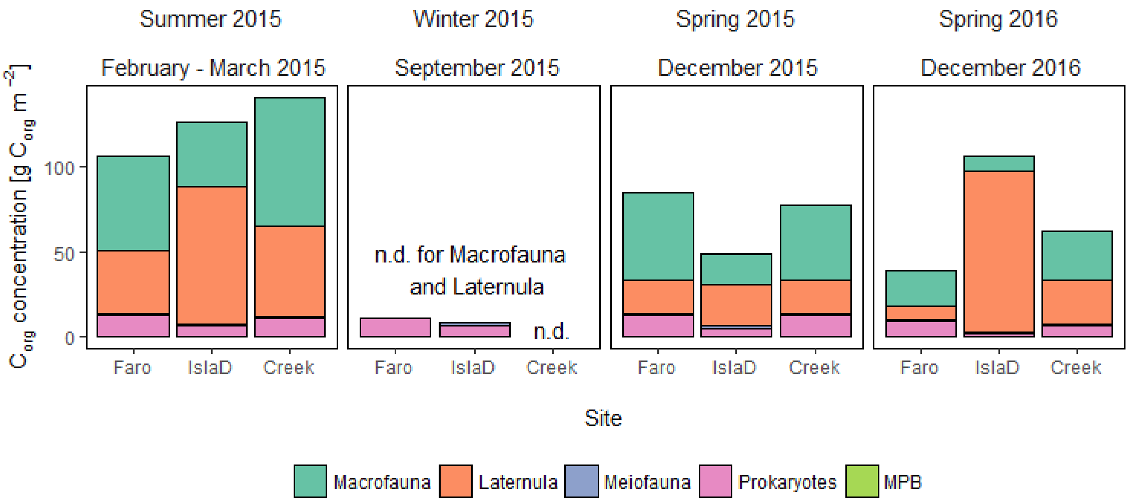

Supplementary Figure 2: Average total benthic biomass in all times and all sites for macrofauna excluding *Laternula elliptica*, *Laternula elliptica*, meiofauna and prokaryotes (integrated over upper 5 cm sediment) and benthic microalgae (MPB; upper 0.5 cm sediment). Statistical differences between sites and seasons for each size group are indicated in Supplementary Table 2. Source data can be found in Supplementary Dataset 5.

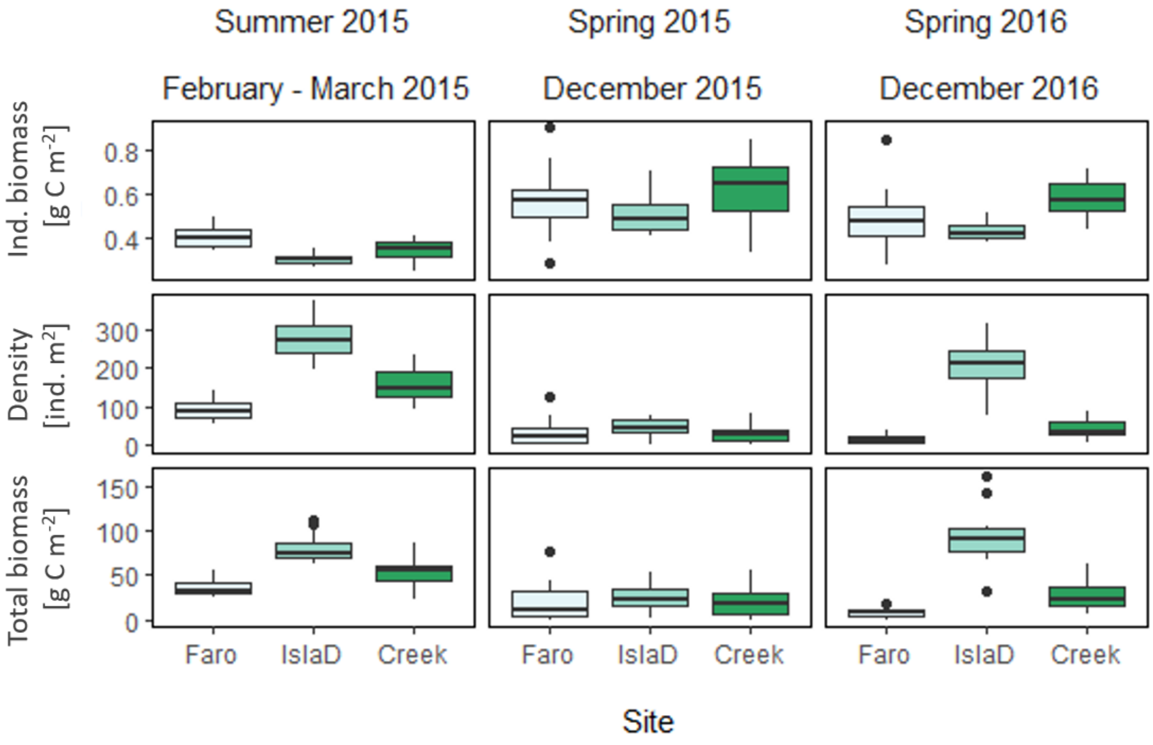

Supplementary Figure 3: **Individual biomass, density and total biomass of the large burrowing bivalve *Laternula elliptica*** in the different sites and seasons. N = 10-16. Boxplots display the median, the hinges represent 25th and 75th quartiles and the whiskers extend to the largest value no further than 1.5 x the inter-quartile range. Outliers are data beyond the end of the whiskers. Statistical differences between sites and seasons are indicated in Supplementary Table 2. Source data can be found in Supplementary Dataset 6.
