# Supplementary material for: Glacial melt disturbance shifts community metabolism of an Antarctic seafloor ecosystem from net autotrophy to heterotrophy
Source: Commun Biol. 2021 Jan 29;4:148. doi: 10.1038/s42003-021-01673-6 (PMC7846736; doi:10.1038/s42003-021-01673-6)
Supplement: Supplementary file 3 — Description of Supplementary Files [file 42003_2021_1673_MOESM3_ESM.pdf]

Description of Additional Supplementary Files

File name: Supplementary Dataset 1

Description: Climatology, Meteorology, Oceanography of Potter Cove 2014-2017 (source data of Figure 2)

File name: Supplementary Dataset 2

Description: Benthic total oxygen exchange, pigments and microalgal biomass of 3 sites in Potter Cove 2015-2016 (source data of Figure 3)

File name: Supplementary Dataset 3

Description: Multivariate dataset of benthic parameters of 3 sites in Potter Cove 2015-2016 (source data of Figure 4 and Supplementary Table 4)

File name: Supplementary Dataset 4

Description: Photosynthetic Active Radiation at 3 sites in Potter Cove 2015-2016 (source data of Supplementary Figure 1)

File name: Supplementary Dataset 5

Description: Average biomass in benthic biotic compartments at 3 sites in Potter Cove 2015-2016 (source data of Supplementary Figure 2)

File name: Supplementary Dataset 6

Description: Individual and total biomass and density of Laternula elliptica at 3 sites in Potter Cove 2015-2016 (source data of Supplementary Figure 3)

File name: Supplementary Dataset 7

Description: Median grain size, silt fraction and porosity at 3 sites in Potter Cove 2015-2016 (source data of Supplementary Table 2).
