# Supplementary material for: Glacial melt disturbance shifts community metabolism of an Antarctic seafloor ecosystem from net autotrophy to heterotrophy
Source: Commun Biol. 2021 Jan 29;4:148. doi: 10.1038/s42003-021-01673-6 (PMC7846736; doi:10.1038/s42003-021-01673-6)
Supplement: Supplementary file 11 — Reporting Summary [file 42003_2021_1673_MOESM11_ESM.pdf]

nature research

Corresponding author(s): Ulrike Braeckman

Last updated by author(s): Nov 30, 2020

Reporting Summary

Nature Research wishes to improve the reproducibility of the work that we publish. This form provides structure for consistency and transparency in reporting. For further information on Nature Research policies, see our Editorial Policies and the Editorial Policy Checklist.

Statistics

For all statistical analyses, confirm that the following items are present in the figure legend, table legend, main text, or Methods section.

n/a	Confirmed	
[ ]	[x]	The exact sample size (*n*) for each experimental group/condition, given as a discrete number and unit of measurement
[ ]	[x]	A statement on whether measurements were taken from distinct samples or whether the same sample was measured repeatedly
[ ]	[x]	The statistical test(s) used AND whether they are one- or two-sided<br>*Only common tests should be described solely by name; describe more complex techniques in the Methods section.*
[x]	[ ]	A description of all covariates tested
[ ]	[x]	A description of any assumptions or corrections, such as tests of normality and adjustment for multiple comparisons
[ ]	[x]	A full description of the statistical parameters including central tendency (e.g. means) or other basic estimates (e.g. regression coefficient) AND variation (e.g. standard deviation) or associated estimates of uncertainty (e.g. confidence intervals)
[ ]	[x]	For null hypothesis testing, the test statistic (e.g. *F*, *t*, *r*) with confidence intervals, effect sizes, degrees of freedom and *P* value noted<br>*Give P values as exact values whenever suitable.*
[x]	[ ]	For Bayesian analysis, information on the choice of priors and Markov chain Monte Carlo settings
[x]	[ ]	For hierarchical and complex designs, identification of the appropriate level for tests and full reporting of outcomes
[x]	[ ]	Estimates of effect sizes (e.g. Cohen's *d*, Pearson's *r*), indicating how they were calculated

Our web collection on statistics for biologists contains articles on many of the points above.

Software and code

Policy information about availability of computer code

Data collection	Data was collected from field samples.
Data analysis	All data analyses were conducted R Statistical Software (version 3.5.2, R Core Team, 2018) using the packages vegan (https://cran.r-project.org/web/packages/vegan/vegan.pdf), car (https://cran.r-project.org/web/packages/car/car.pdf) and glmnet (https://cran.r-project.org/web/packages/glmnet/glmnet.pdf).

For manuscripts utilizing custom algorithms or software that are central to the research but not yet described in published literature, software must be made available to editors and reviewers. We strongly encourage code deposition in a community repository (e.g. GitHub). See the Nature Research guidelines for submitting code & software for further information.

Data

Policy information about availability of data

All manuscripts must include a data availability statement. This statement should provide the following information, where applicable:

- Accession codes, unique identifiers, or web links for publicly available datasets
- A list of figures that have associated raw data
- A description of any restrictions on data availability

Data will be made publicly available at PANGAEA Data Publisher for Earth & Environmental Science (www.pangaea.de) upon publication of the manuscript.

Field-specific reporting

Please select the one below that is the best fit for your research. If you are not sure, read the appropriate sections before making your selection.

☐ Life sciences ☐ Behavioural & social sciences ☒ Ecological, evolutionary & environmental sciences

For a reference copy of the document with all sections, see nature.com/documents/nr-reporting-summary-flat.pdf

Life sciences study design

All studies must disclose on these points even when the disclosure is negative.

Sample size	*Describe how sample size was determined, detailing any statistical methods used to predetermine sample size OR if no sample-size calculation was performed, describe how sample sizes were chosen and provide a rationale for why these sample sizes are sufficient.*
Data exclusions	*Describe any data exclusions. If no data were excluded from the analyses, state so OR if data were excluded, describe the exclusions and the rationale behind them, indicating whether exclusion criteria were pre-established.*
Replication	*Describe the measures taken to verify the reproducibility of the experimental findings. If all attempts at replication were successful, confirm this OR if there are any findings that were not replicated or cannot be reproduced, note this and describe why.*
Randomization	*Describe how samples/organisms/participants were allocated into experimental groups. If allocation was not random, describe how covariates were controlled OR if this is not relevant to your study, explain why.*
Blinding	*Describe whether the investigators were blinded to group allocation during data collection and/or analysis. If blinding was not possible, describe why OR explain why blinding was not relevant to your study.*

Behavioural & social sciences study design

All studies must disclose on these points even when the disclosure is negative.

Study description	*Briefly describe the study type including whether data are quantitative, qualitative, or mixed-methods (e.g. qualitative cross-sectional, quantitative experimental, mixed-methods case study).*
Research sample	*State the research sample (e.g. Harvard university undergraduates, villagers in rural India) and provide relevant demographic information (e.g. age, sex) and indicate whether the sample is representative. Provide a rationale for the study sample chosen. For studies involving existing datasets, please describe the dataset and source.*
Sampling strategy	*Describe the sampling procedure (e.g. random, snowball, stratified, convenience). Describe the statistical methods that were used to predetermine sample size OR if no sample-size calculation was performed, describe how sample sizes were chosen and provide a rationale for why these sample sizes are sufficient. For qualitative data, please indicate whether data saturation was considered, and what criteria were used to decide that no further sampling was needed.*
Data collection	*Provide details about the data collection procedure, including the instruments or devices used to record the data (e.g. pen and paper, computer, eye tracker, video or audio equipment) whether anyone was present besides the participant(s) and the researcher, and whether the researcher was blind to experimental condition and/or the study hypothesis during data collection.*
Timing	*Indicate the start and stop dates of data collection. If there is a gap between collection periods, state the dates for each sample cohort.*
Data exclusions	*If no data were excluded from the analyses, state so OR if data were excluded, provide the exact number of exclusions and the rationale behind them, indicating whether exclusion criteria were pre-established.*
Non-participation	*State how many participants dropped out/declined participation and the reason(s) given OR provide response rate OR state that no participants dropped out/declined participation.*
Randomization	*If participants were not allocated into experimental groups, state so OR describe how participants were allocated to groups, and if allocation was not random, describe how covariates were controlled.*

Ecological, evolutionary & environmental sciences study design

All studies must disclose on these points even when the disclosure is negative.

Study description	In this study, we investigated benthic community metabolism based on net production and respiration at three sites with differing glacial melt impact in Potter Cove, King George Island/Isla 25 de Mayo (Fig. 1, Table S1), during summer, winter and spring 2015 (strong El Niño year) and in spring 2016 (weak La Niña year) and examined environmental drivers in climatology, cryosphere and oceanography that relate to glacial melt conditions.
Research sample	Meteorology: air temperature, wind speed and direction<br>Sea ice cover: number of days with sea ice in the cove<br>Oceanography:<br>measurements of water column temperature, salinity, SPM, chl a.<br>Benthic samples:<br>-measurements of oxygen concentration in overlying water of benthic chambers<br>-sediment samples for environmental variables<br>-sediment samples for infauna characterisation
Sampling strategy	Oceanography: The oceanographic sampling is part of a long-term monitoring program that started in 1991. The chosen long-term monitoring station is located in inner Potter Cove (E1, see Schloss et al. 2012 doi: 10.1016/j.jmarsys.2011.10.006).<br>Benthic sample sizes were based on best practices obtained from previous soft sediment ecological research by the research team (Pasotti et al. 2015 doi: 10.1111/maec.12179 and Hoffmann et al. 2018 https://doi.org/10.1371/journal.pone.0207917) and taken into account logistic feasibility (number of samples taken during a 20 min dive in cold water (-1° - +2°C).
Data collection	Meteorology: Air temperature, wind speed and direction, were measured every 3 hours by the Servicio Meteorológico Nacional (SMN) of the Argentine Air Force at Carlini Station.<br>Sea ice cover: The period and the annual number of days with ice cover was calculated for Potter Cove using daily photographic observations of the presence or absence of ice cover between 2014 to 2017.<br>Oceanographic data:<br>A Sea-Bird SBE CTD (Sea-Bird Electronics Inc., USA) was used for data on seawater temperature and conductivity (transformed in salinity). Average Sea Surface Temperature (SST) and salinity were extracted from the upper meter from the long-term monitoring station in inner Potter Cove (E1, see Schloss et al. 2012 doi: 10.1016/j.jmarsys.2011.10.006). Bottom water temperature (6-9 m water depth) was recorded with HOBO Pendant loggers (Onset, Bourne, USA) during in situ benthic chamber deployments (see 'Community metabolism measurements'). Water samples for the determination of SPM and chlorophyll-a were collected at four depths (i.e., 0, 5, 10 and 20 m) at the long-term monitoring station in inner Potter Cove with 4.7 L Niskin bottles.<br>Benthic data:<br>For the determination of sediment properties and biogenic sediment compounds, sediment was sampled with 3.6 cm diameter plexiglass cores in three to five replicates by SCUBA divers. Prokaryotic, benthic microalgal and meiofaunal counts were also determined from these sediment samples. Ulrike Braeckman, Francesca Pasotti, Ralf Hoffmann and specifically trained overwintering scientist Francisco Ferrer followed the same protocol for further sediment sample processing. Benthic macrofauna was sampled with a Van Veen grab (530 cm² surface area), deployed by SCUBA divers from a Zodiac. At each location, three to four sediment samples were sieved over a 1 mm mesh. Macrofauna organisms were identified to at least family level by Nene Lefaible. The Van Veen grab sampling results in a strong underestimation of the density of the large Antarctic bivalve Laternula elliptica. To estimate Laternula elliptica densities, two transects of eight grids (45 cm x 45 cm) were randomly placed on the seafloor of each location by scientific divers Francesca Pasotti, Ralf Hoffmann and Anders Torstensson and photos were taken (Nikon D750 with a rectilinear Nikon 16–35 mm lens in a Nauticam underwater housing and two Inon Z-240 strobes).<br>Community metabolism measurements:<br>Scientific divers Francesca Pasotti, Ralf Hoffmann, Anders Torstensson and specifically trained overwintering divers sampled the overlying water of the benthic chambers. The water samples were further processed and oxygen concentration was measured by Winkler titration by Ulrike Braeckman, Francesca Pasotti, Ralf Hoffmann and specifically trained overwintering scientist Francisco Ferrer.
Timing and spatial scale	Meteorology: measurements every 3 hours<br>Sea ice cover: daily photographic observations<br>Oceanography: The water column in Potter Cove is sampled weekly in summer and bi-weekly in winter, whenever the meteorological conditions allow it at several sampling stations in the Cove.<br>Benthic sampling was conducted in February - March 2015 (austral summer), September 2015 (austral winter, under the ice), December 2015 (austral spring) and December 2016 (austral spring). See supplementary information (Table S1) for exact dates and locations.
Data exclusions	Benthic sampling: Three values of NCM and two values of CR were omitted from further analysis because of technical issues during incubation (one transparent chamber at Isla D in summer 2015, one transparent chamber at Isla D and one at Faro in spring 2015, one dark chamber at Faro and one at Creek in spring 2016).
Reproducibility	The data were not collected from laboratory experiments, but were collected in situ. The methods to collect the data were successfully repeated in different seasons.
Randomization	Benthic sampling: scientific divers randomly deployed benthic chambers and took random sediment cores on each benthic location.

Blinding | Technicians and students that processed the samples were not familiar with the sampling codes.

Did the study involve field work? ☒ Yes ☐ No

Field work, collection and transport

Field conditions | Measurements of benthic community metabolism and sampling of benthic communities and their habitat was conducted in February - March 2015 (austral summer), September 2015 (austral winter, under the ice), December 2015 (austral spring) and December 2016 (austral spring). Meteorological, glaciological and oceanographic conditions during the benthic community metabolism measurements and benthic sampling are shown in Figure 2 and are broadly described in the manuscript.

Location | Potter Cove (62° 14' S 58° 38' W), King George Island, West Antarctic Peninsula.
Water column samples: E1, 62.238°S; 58.689°W (see Schloss et al. 2012 doi:10.1016/j.jmarsys.2011.10.006).
Benthic samples: 6-9m water depth, at three locations < 1km from another. Specific coordinates of benthic sampling are available in the supplementary information (Table S1).

Access & import/export | Sampling locations were accessed from the land-based Carlini station by Zodiac and scientific divers (benthic sampling) and/or scientists (water column sampling) during summer or with sledges over the sea ice during winter.

Disturbance | Scientific divers took utmost care to not disturb the sediment surface while deploying benthic chambers or taking sediment cores for benthic habitat characterisation. A small Van Veen grab (530 cm^2 = 0.053 m^2 surface area instead of the regular 0.1m^2 surface area) was used for collection of infauna samples in order to limit the sampling impact on the benthic ecosystem.

Reporting for specific materials, systems and methods

We require information from authors about some types of materials, experimental systems and methods used in many studies. Here, indicate whether each material, system or method listed is relevant to your study. If you are not sure if a list item applies to your research, read the appropriate section before selecting a response.

Materials & experimental systems

n/a	Involved in the study	
☒	☐	Antibodies
☒	☐	Eukaryotic cell lines
☒	☐	Palaeontology and archaeology
☐	☒	Animals and other organisms
☒	☐	Human research participants
☒	☐	Clinical data
☒	☐	Dual use research of concern

Methods

n/a	Involved in the study	
☒	☐	ChIP-seq
☒	☐	Flow cytometry
☒	☐	MRI-based neuroimaging

Antibodies

Antibodies used | *Describe all antibodies used in the study; as applicable, provide supplier name, catalog number, clone name, and lot number.*

Validation | *Describe the validation of each primary antibody for the species and application, noting any validation statements on the manufacturer's website, relevant citations, antibody profiles in online databases, or data provided in the manuscript.*

Eukaryotic cell lines

Policy information about cell lines

Cell line source(s) | *State the source of each cell line used.*

Authentication | *Describe the authentication procedures for each cell line used OR declare that none of the cell lines used were authenticated.*

Mycoplasma contamination | *Confirm that all cell lines tested negative for mycoplasma contamination OR describe the results of the testing for mycoplasma contamination OR declare that the cell lines were not tested for mycoplasma contamination.*

Commonly misidentified lines (See ICLAC register) | *Name any commonly misidentified cell lines used in the study and provide a rationale for their use.*

Palaeontology and Archaeology

Specimen provenance | *Provide provenance information for specimens and describe permits that were obtained for the work (including the name of the issuing authority, the date of issue, and any identifying information).*

Specimen deposition | *Indicate where the specimens have been deposited to permit free access by other researchers.*

Dating methods | *If new dates are provided, describe how they were obtained (e.g. collection, storage, sample pretreatment and measurement), where they were obtained (i.e. lab name), the calibration program and the protocol for quality assurance OR state that no new dates are provided.*

☐ Tick this box to confirm that the raw and calibrated dates are available in the paper or in Supplementary Information.

Ethics oversight | *Identify the organization(s) that approved or provided guidance on the study protocol, OR state that no ethical approval or guidance was required and explain why not.*

Note that full information on the approval of the study protocol must also be provided in the manuscript.

Animals and other organisms

Policy information about studies involving animals; ARRIVE guidelines recommended for reporting animal research

Laboratory animals	The study did not involve laboratory animals.
Wild animals	Invertebrate macrofauna was collected with a small Van Veen grab (530 cm^2) and killed using a 4% formaldehyde-seawater solution enabling subsequent identification using stereomicroscopy.
Field-collected samples	The study did not involve experimentation with field-collected samples.
Ethics oversight	No ethical approval was required for the collection of the studied invertebrates.

Note that full information on the approval of the study protocol must also be provided in the manuscript.

Human research participants

Policy information about studies involving human research participants

Population characteristics	*Describe the covariate-relevant population characteristics of the human research participants (e.g. age, gender, genotypic information, past and current diagnosis and treatment categories). If you filled out the behavioural & social sciences study design questions and have nothing to add here, write "See above."*
Recruitment	*Describe how participants were recruited. Outline any potential self-selection bias or other biases that may be present and how these are likely to impact results.*
Ethics oversight	*Identify the organization(s) that approved the study protocol.*

Note that full information on the approval of the study protocol must also be provided in the manuscript.

Clinical data

Policy information about clinical studies

All manuscripts should comply with the ICMJEguidelines for publication of clinical research and a completedCONSORT checklist must be included with all submissions.

Clinical trial registration	*Provide the trial registration number from ClinicalTrials.gov or an equivalent agency.*
Study protocol	*Note where the full trial protocol can be accessed OR if not available, explain why.*
Data collection	*Describe the settings and locales of data collection, noting the time periods of recruitment and data collection.*
Outcomes	*Describe how you pre-defined primary and secondary outcome measures and how you assessed these measures.*

Dual use research of concern

Policy information about dual use research of concern

Hazards

Could the accidental, deliberate or reckless misuse of agents or technologies generated in the work, or the application of information presented in the manuscript, pose a threat to:

No	Yes	
☐	☐	Public health
☐	☐	National security
☐	☐	Crops and/or livestock
☐	☐	Ecosystems
☐	☐	Any other significant area

Experiments of concern

Does the work involve any of these experiments of concern:

No	Yes	
☐	☐	Demonstrate how to render a vaccine ineffective
☐	☐	Confer resistance to therapeutically useful antibiotics or antiviral agents
☐	☐	Enhance the virulence of a pathogen or render a nonpathogen virulent
☐	☐	Increase transmissibility of a pathogen
☐	☐	Alter the host range of a pathogen
☐	☐	Enable evasion of diagnostic/detection modalities
☐	☐	Enable the weaponization of a biological agent or toxin
☐	☐	Any other potentially harmful combination of experiments and agents

ChIP-seq

Data deposition

☐ Confirm that both raw and final processed data have been deposited in a public database such as GEO.

☐ Confirm that you have deposited or provided access to graph files (e.g. BED files) for the called peaks.

Data access links<br>*May remain private before publication.*	*For "Initial submission" or "Revised version" documents, provide reviewer access links. For your "Final submission" document, provide a link to the deposited data.*
Files in database submission	*Provide a list of all files available in the database submission.*
Genome browser session<br>(e.g. UCSC)	*Provide a link to an anonymized genome browser session for "Initial submission" and "Revised version" documents only, to enable peer review. Write "no longer applicable" for "Final submission" documents.*

Methodology

Replicates	*Describe the experimental replicates, specifying number, type and replicate agreement.*
Sequencing depth	*Describe the sequencing depth for each experiment, providing the total number of reads, uniquely mapped reads, length of reads and whether they were paired- or single-end.*
Antibodies	*Describe the antibodies used for the ChIP-seq experiments; as applicable, provide supplier name, catalog number, clone name, and lot number.*
Peak calling parameters	*Specify the command line program and parameters used for read mapping and peak calling, including the ChIP, control and index files used.*
Data quality	*Describe the methods used to ensure data quality in full detail, including how many peaks are at FDR 5% and above 5-fold enrichment.*
Software	*Describe the software used to collect and analyze the ChIP-seq data. For custom code that has been deposited into a community repository, provide accession details.*

Flow Cytometry

Plots

Confirm that:

- [ ] The axis labels state the marker and fluorochrome used (e.g. CD4-FITC).
- [ ] The axis scales are clearly visible. Include numbers along axes only for bottom left plot of group (a 'group' is an analysis of identical markers).
- [ ] All plots are contour plots with outliers or pseudocolor plots.
- [ ] A numerical value for number of cells or percentage (with statistics) is provided.

Methodology

Sample preparation	*Describe the sample preparation, detailing the biological source of the cells and any tissue processing steps used.*
Instrument	*Identify the instrument used for data collection, specifying make and model number.*
Software	*Describe the software used to collect and analyze the flow cytometry data. For custom code that has been deposited into a community repository, provide accession details.*
Cell population abundance	*Describe the abundance of the relevant cell populations within post-sort fractions, providing details on the purity of the samples and how it was determined.*
Gating strategy	*Describe the gating strategy used for all relevant experiments, specifying the preliminary FSC/SSC gates of the starting cell population, indicating where boundaries between "positive" and "negative" staining cell populations are defined.*

- [ ] Tick this box to confirm that a figure exemplifying the gating strategy is provided in the Supplementary Information.

Magnetic resonance imaging

Experimental design

Design type	*Indicate task or resting state; event-related or block design.*
Design specifications	*Specify the number of blocks, trials or experimental units per session and/or subject, and specify the length of each trial or block (if trials are blocked) and interval between trials.*
Behavioral performance measures	*State number and/or type of variables recorded (e.g. correct button press, response time) and what statistics were used to establish that the subjects were performing the task as expected (e.g. mean, range, and/or standard deviation across subjects).*

Acquisition

Imaging type(s)	*Specify: functional, structural, diffusion, perfusion.*
Field strength	*Specify in Tesla*
Sequence & imaging parameters	*Specify the pulse sequence type (gradient echo, spin echo, etc.), imaging type (EPI, spiral, etc.), field of view, matrix size, slice thickness, orientation and TE/TR/flip angle.*
Area of acquisition	*State whether a whole brain scan was used OR define the area of acquisition, describing how the region was determined.*

Diffusion MRI ☐ Used ☐ Not used

Preprocessing

Preprocessing software	*Provide detail on software version and revision number and on specific parameters (model/functions, brain extraction, segmentation, smoothing kernel size, etc.).*
Normalization	*If data were normalized/standardized, describe the approach(es): specify linear or non-linear and define image types used for transformation OR indicate that data were not normalized and explain rationale for lack of normalization.*
Normalization template	*Describe the template used for normalization/transformation, specifying subject space or group standardized space (e.g. original Talairach, MNI305, ICBM152) OR indicate that the data were not normalized.*
Noise and artifact removal	*Describe your procedure(s) for artifact and structured noise removal, specifying motion parameters, tissue signals and physiological signals (heart rate, respiration).*

Volume censoring | *Define your software and/or method and criteria for volume censoring, and state the extent of such censoring.*

Statistical modeling & inference

Model type and settings | *Specify type (mass univariate, multivariate, RSA, predictive, etc.) and describe essential details of the model at the first and second levels (e.g. fixed, random or mixed effects; drift or auto-correlation).*

Effect(s) tested | *Define precise effect in terms of the task or stimulus conditions instead of psychological concepts and indicate whether ANOVA or factorial designs were used.*

Specify type of analysis: ☐ Whole brain ☐ ROI-based ☐ Both

Statistic type for inference
(See Eklund et al. 2016) | *Specify voxel-wise or cluster-wise and report all relevant parameters for cluster-wise methods.*

Correction | *Describe the type of correction and how it is obtained for multiple comparisons (e.g. FWE, FDR, permutation or Monte Carlo).*

Models & analysis

n/a	Involved in the study
☐	☐ Functional and/or effective connectivity
☐	☐ Graph analysis
☐	☐ Multivariate modeling or predictive analysis

Functional and/or effective connectivity | *Report the measures of dependence used and the model details (e.g. Pearson correlation, partial correlation, mutual information).*

Graph analysis | *Report the dependent variable and connectivity measure, specifying weighted graph or binarized graph, subject- or group-level, and the global and/or node summaries used (e.g. clustering coefficient, efficiency, etc.).*

Multivariate modeling and predictive analysis | *Specify independent variables, features extraction and dimension reduction, model, training and evaluation metrics.*
